# Characterization of interactions between hepatitis C virus NS5B polymerase, annexin A2 and RNA – effects on NS5B catalysis and allosteric inhibition

**DOI:** 10.1186/s12985-017-0904-4

**Published:** 2017-12-11

**Authors:** Sara M. Ø. Solbak, Eldar Abdurakhmanov, Anni Vedeler, U. Helena Danielson

**Affiliations:** 1 Department of Chemistry – BMC, Uppsala University, Uppsala, Sweden; 2 Department of Biomedicine, University of Bergen, Bergen, Norway; 3 Science for Life Laboratory, Uppsala University, Uppsala, Sweden

**Keywords:** Hepatitis C virus, NS5B, Annexin A2, RNA, allosteric inhibition, filibuvir, surface plasmon resonance, Hepatocellular carcinoma

## Abstract

**Background:**

Direct acting antivirals (DAAs) provide efficient hepatitis C virus (HCV) therapy and clearance for a majority of patients, but are not available or effective for all patients. They risk developing HCV-induced hepatocellular carcinoma (HCC), for which the mechanism remains obscure and therapy is missing. Annexin A2 (AnxA2) has been reported to co-precipitate with the non-structural (NS) HCV proteins NS5B and NS3/NS4A, indicating a role in HCC tumorigenesis and effect on DAA therapy.

**Methods:**

Surface plasmon resonance biosensor technology was used to characterize direct interactions between AnxA2 and HCV NS5B, NS3/NS4 and RNA, and the subsequent effects on catalysis and inhibition.

**Results:**

No direct interaction between AnxA2 and NS3/NS4A was detected, while AnxA2 formed a slowly dissociating, high affinity (*K*_D_ = 30 nM), complex with NS5B, decreasing its catalytic activity and affinity for the allosteric inhibitor filibuvir. The RNA binding of the two proteins was independent and AnxA2 and NS5B interacted with different RNAs in ternary complexes of AnxA2:NS5B:RNA, indicating specific preferences.

**Conclusions:**

The complex interplay revealed between NS5B, AnxA2, RNA and filibuvir, suggests that AnxA2 may have an important role for the progression and treatment of HCV infections and the development of HCC, which should be considered also when designing new allosteric inhibitors.

**Electronic supplementary material:**

The online version of this article (10.1186/s12985-017-0904-4) contains supplementary material, which is available to authorized users.

## Background

Although direct acting antivirals (DAAs) have improved the therapeutic options for hepatitis C virus (HCV) infections radically, there is still a need for continued research on disease mechanisms and new therapeutic strategies, as many patients do not have access to DAAs or are infected with genotypes insensitive to current drugs [1].

The formation of the HCV replication complex is known to require various viral non-structural (NS) proteins as well as host cellular proteins. In addition, the HCV replication machinery is associated with a specific membrane alteration, “the membranous web”, derived from endoplasmic reticulum (ER) membranes. The NS proteins involved in replication (NS3, NS4A, NS5A, NS5B) are anchored to the membrane via several integral or peripheral membrane binding domains [2]. As our efforts are currently focused on understanding the interplay between HCV and host cellular proteins, reports that the host cellular protein AnxA2 is associated with the non-structural HCV proteins in the replication complex [3–5] attracted our attention. AnxA2 belongs to the calcium- and phospholipid-binding protein family of annexins [6, 7] and is a host cellular marker found aberrantly expressed in various malignant tumors (colon, lung, gastric, oesophageal, and breast) [8–12] including hepatocellular carcinoma (HCC) [13–17].

AnxA2 has been isolated from the HCV replication complex and shown to be recruited by NS5A to the HCV replication site [5]. By suppression of AnxA2 expression, it has been established that AnxA2 reduces HCV titers and has a direct role in HCV infections [3]. More specifically, it has been established that AnxA2 interacts with NS3/NS4A, NS5A [3, 4] and NS5B [3], and that it co-localizes with these proteins in the perinuclear region. Interestingly, Ser25 phosphorylated AnxA2 is associated with translationally inactive messenger ribonucleoprotein (mRNP) complexes in the perinuclear region of the host cells [18]. Since NS3/NS4A is a membrane anchored protein and AnxA2 is known as a lipid raft-associated scaffold protein, it has been postulated that AnxA2 assists in the formation of HCV replication complexes in the lipid rafts [3].

Although AnxA2 has been demonstrated to co-precipitate with the membrane-anchored NS5B and NS3/NS4A proteins [3], the nature of these interactions has not been investigated previously. NS5B is a 68 kDa RNA-polymerase and NS3/NS4A is a 67 kDa protease-helicase. Both enzymes have central roles in replication, and have been successfully exploited as targets for HCV drugs. In order to better understand the interactions between AnxA2 and these viral enzymes, we have used surface plasmon resonance (SPR) biosensor technology to characterize the details of the interactions, as well as how the binding of AnxA2 influences the interaction between NS5B and RNA and an allosteric polymerase inhibitor. To elucidate the importance of the RNA-binding ability of AnxA2 on its interaction with NS5B and its effects on polymerase activity, a mutant form of AnxA2 (mAnxA2) that is unable to bind RNA was also used [19].

This study reveals that AnxA2 binds directly to NS5B and reduces its polymerase activity, thus providing a better understanding of how AnxA2 may be involved in the HCV life cycle and with implications for the design of novel allosteric inhibitors of NS5B.

## Methods

### Protein expression and purification

The ectodomain of NS5B (NS5BΔ21) from genotype 1b was produced as earlier described, with the details of collection of blood samples, viral RNA extraction, cDNA formation, and cloning of NS5B 1b DNA into an expression vector published in [20] and methods for protein expression and purification published in [21]. HCV NS3 and HCV NS3-4A genotype 1a were produced as previously described [20].

Bovine AnxA2 and an engineered variant containing mutations in helix C and in the CD loop (Ser substitutions of Lys308, Lys309 and Lys310 in helix C, Lys313 in the CD loop and Tyr317 and Gln321 in helix D) (mAnxA2) were expressed and purified as previously described [19].

### RNA synthesis

The 31-mer RNA (5’CGAUACUCCCUUUAUAUAACCAUCAAUCGCC 3′) used in SPR polymerase assay [22], was synthesized as previously described [23]. Briefly, the DNA oligos:

5′ ATTCGTTAATACGACTCACTATAGGG 3′ and. 

5′ GGCGATTGATGGTTATATAAAGGGAGTATCGCCCTATAGTGAGTCGTATTA 3′.

were used as a template for the 31 bp RNA synthesis. The primers were annealed by heating to 100 °C for 1 min in 50 mM Tris-HCl pH 7.4 and 100 mM KCl buffer. The RNA was synthesized using Riboprobe T7 kit (Promega, Fitchburg, WI, USA) according to the manufacturer’s instructions. The synthesized RNA was purified with standard phenol/chlorophorm extraction procedure. Unincorporated nucleotides were removed using PD SpinTrap G-25 spin columns (GE Healthcare, Uppsala, Sweden).

The 8-mer RNA (5’GGG GAU UG-3′) used in interaction studies with AnxA2 and NS5B was purchased from Eurofins Genomics.

### Surface plasmon resonance biosensor analysis

SPR measurements were performed at 25 °C using a Biacore S51 or T200 instrument and the data was analyzed using Biacore T200 evaluation software version 1.0 (GE Healthcare, Uppsala, Sweden). Experiments were performed using HBS-EP running buffer (0.01 M 4-(2-hydroxyethyl)-1-piperazineethanesulfonic acid (HEPES) pH 7.4, 0.15 M NaCl, 3 mM ethylene diamine tetra acetic acid (EDTA), 0.005% *v*/v Surfactant P20) in the presence of 70 μM Ca^2+^ and 1 mM Mg^2+^, using a flow rate of 30 μl/min. AnxA2 or NS5B (genotype 1b BK) were immobilized on a CM5 chip by standard amine coupling using 5 mM maleate buffer pH 6 and NaAc buffer pH 6, respectively, as immobilization buffer. Multi cycle experiments with regeneration after each injection (with 2 M NaCl and 2 M MgCl_2_), or single cycle kinetic experiments, without regeneration between the injections, were performed with four or five concentrations of the analyte in HBS-EP running buffer. In experiments investigating the interplay between AnxA2, NS5B and RNA, AnxA2 was immobilized as above, and either 8-mer RNA or NS5B was injected to form a stable binary complex, followed by injection of the third interaction partner.

The sensorgrams were corrected for buffer bulk effects and unspecific binding of the samples to the chip matrix by blank and reference surface subtraction (subtraction of inactivated and deactivated flow cell channel or where NS5B was immobilized and surface inactivated by 3 × 30 s injections of 6 M guanidine-HCl).

The association rates (*k*_a_), dissociation rates (*k*_d_), dissociation constants (K_D_) and maximum binding responses (R_max_) were estimated by global non-linear regression analysis and a reversible 1-step interaction reaching steady-state at the end of the analyte injection or by fitting the sensorgram to a reversible 1-step interaction model. The analysis was based on report points taken at the end of analyte injections even if steady state had not been reached. In cases where the interaction mechanism was not established, the analysis is purely qualitative and any estimated K_D_-values are termed “apparent” (K_D_^app^), only useful for discussions on the possible order of magnitude of affinities.

### Enzyme activity assay

A continuous de novo HCV NS5B polymerase assay, using surface plasmon resonance biosensor technology (Biacore T200, GE Healthcare, Uppsala, Sweden), was developed, as illustrated in Fig. 3a (from [23]). Streptavidin (Sigma-Aldrich, St. Louis, MO, USA) was diluted to 100 μg/ml in the buffer containing 10 mM NaAc pH 5, 0.1 mM EDTA, 1 mM NaCl, 1 mM dithiothreitol (DTT) and immobilized on the CM5 chip surface by standard amine coupling procedure, resulting in 4500 Refractive units (RU). The surface was washed 3 times with conditioning solution (1 M NaCl, 50 mM NaOH) to remove excess unbound streptavidin. Subsequently, about 500–700 RU of biotinylated ssDNA oligo diluted to 660 nM in the running buffer (10 mM Tris-HCl pH 7.4, 50 mM NaCl, 1 mM EDTA, 1 mM β-mercaptoethanol and 0.005% Tween 20) was captured by streptavidin-biotin interaction. This DNA oligo has a complementary sequence to the in vitro synthesized single-stranded (ss) 31-mer RNA, which was hybridized in the next step to an approximate level of 200–300 RU. The ss31-mer RNA, an oligomer with at sequence originally designed for a de novo polymerase assay [22], was diluted in the same buffer as ssDNA to a final concentration of 20 ng/μl. The NS5B Δ21 polymerase and/or NS5B Δ21 supplemented with 750 μM ribo nucleotide triphosphates (rNTPs) (Promega, Fitchburg, WI, USA) were injected over the surface containing the DNA/RNA hybrid and over the reference surface with immobilized streptavidin. The effect of AnxA2 or mAnxA2 on polymerase activity was tested by adding 100 nM or 200 nM of AnxA2/mAnxA2 to the NS5B/rNTPs mixture. The assay was performed using HBS-EP running buffer (0.01 M HEPES pH 7.4, 0.15 M NaCl, 3 mM EDTA, 0.005% *v*/v Surfactant P20) in the presence of 70 μM Ca^2+^ and 1 mM Mg^2+^. The data was analyzed using Biacore T200 evaluation software version 1.0 (GE Healthcare). The NS3 based enzyme activity assay was performed according to the protocol described previously [24].

## Results

### Establishing direct AnxA2-NS5B interaction

**Fig. 1:**
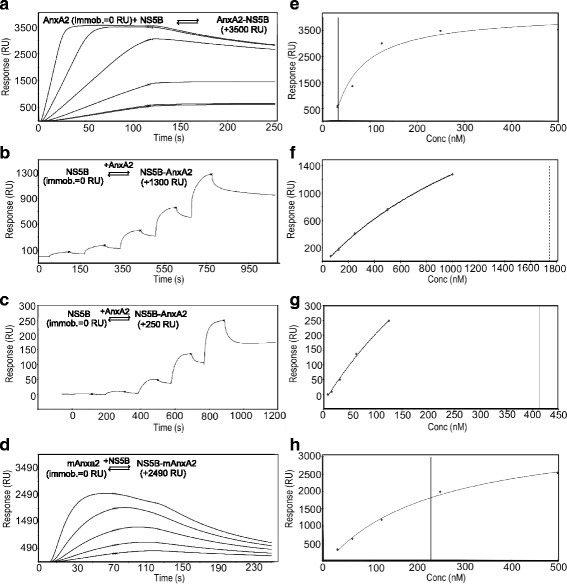
Interactions between AnxA2 and NS5B demonstrated in real-time by SPR biosensor technology. **a** 31 to 500 nM NS5B injected over immobilized AnxA2 (3595 RU), using an activated and inactivated blank surface as reference. **b** 63 to 1000 nM AnxA2 injected over immobilized NS5B (2634 RU). **c** 7.8 to 125 nM AnxA2 injected over immobilized NS5B (12,400 RU). The reference surface contained immobilized NS5B (11,765 RU) inactivated by injections of 6 M guanidine-HCl. **d** 31 to 500 nM NS5B injected over immobilized mAnxA2 (unable to bind RNA) (1041 RU). An activated and inactivated blank surface was used as reference. Experiments with immobilized AnxA2 wt and mAnxA2 (**a** and **d**, respectively) included regeneration of the surface between analyte injections, while experiments with immobilized NS5B (**b** and **c**) were performed without regeneration of the surface between injections. **e**-**h** Steady state analysis of experiments **a**-**d** was based on report points taken at the end of the injection although steady state was not reached in all report points. Vertical lines on steady-state analysis plots indicate K_D_ values (i.e. concentration resulting in 50% saturation)

To investigate a possible direct interaction between AnxA2 and NS5B polymerase, AnxA2 was immobilized on an SPR sensor chip and NS5B was run as analyte (Fig. 1a). The experiment demonstrated that AnxA2 and NS5B formed a stable complex with a very slow rate of dissociation. The interaction between the two proteins had to be actively broken for the signal to return to baseline before a new sample of analyte could be injected. A regeneration solution of high ionic strength (2 M NaCl and 2 M MgCl_2_) was found to be suitable.

**Fig. 2:**
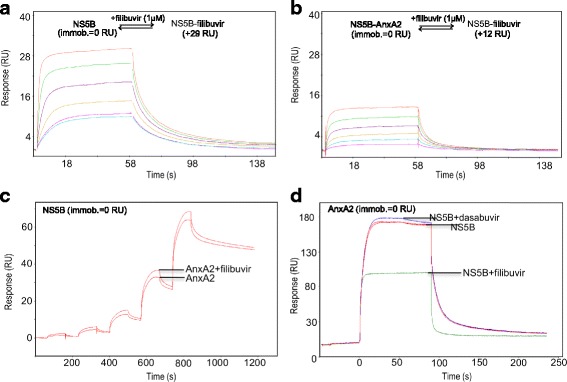
Effect of allosteric NS5B inhibitors on AnxA2-NS5B interaction. Sensorgrams for the injection of **a** 63 to 1000 nM filibuvir over immobilized NS5B (12,400 RU), and **b** 63 to 1000 nM filibuvir after an injection of 125 nM AnxA2. The reference cell contained immobilized NS5B (11,765 RU) inactivated by injections of 6 M guanidine-HCl. All sensorgrams are blank injection and reference surface subtracted. Sensorgrams for **c** interaction between 63 to 1000 nM AnxA2 (bottom curve) and injected simultaneously with 500 nM filibuvir (top curve) over immobilized NS5B (2634 RU), and **d** interaction between 200 nM NS5B (red) injected simultaneously with 1 mM of filibuvir (green) or dasabuvir (blue) over immobilized AnxA2 (3785 RU)

To confirm the interaction, the reverse experiment was performed, i.e. with NS5B immobilized on the chip and AnxA2 used as analyte. Moreover, to avoid artefacts potentially induced by a regeneration solution, single cycle kinetic (SCK) experiments were performed. This experimental approach confirmed the binding of AnxA2 to NS5B (Fig. 1b). To eliminate the possibility that the binding to NS5B was unspecific, the interaction was also analyzed using a reference flow cell where NS5B was first immobilized and subsequently inactivated (denatured) by multiple injections of 6 M guanidine-HCl (Fig. 1c). Successful inactivation of NS5B in the reference flow cell was confirmed by showing that the HCV polymerase inhibitor filibuvir did not interact with the denatured protein in the reference cell. Similar SPR sensorgrams of the interaction of filibuvir to immobilized NS5B was achieved for the experiment with an inactivated NS5B immobilized on the reference cell (Fig. 2a) compared to the experiment with a blank reference cell. The interaction seen for AnxA2 in the assay where unspecific binding was subtracted, confirmed a strong interaction between NS5B and AnxA2.

The interaction between AnxA2 and NS5B may be influenced by the fact that both are viral RNA-binding proteins. For example, it is possible that AnxA2 bound to host mRNA is not able to bind NS5B or that AnxA2 bound to NS5B is not able to bind RNA, indicating an overlap of the binding sites. In addition, if mAnxA2 interacts with NS5B, this would exclude helices C-D as the site of interaction between the two proteins. The non-RNA binding engineered AnxA2 variant, mAnxA2, was therefore also tested for its ability to interact with NS5B (Fig. 1d). Although the experiment confirmed a strong interaction between NS5B and immobilized mAnxA2, it was weaker than that observed between NS5B and *wild type* (*wt*) AnxA2 and dissociated more rapidly (Fig. 1a). The non-ideal shape of the sensorgrams indicated that the interaction was not stable, potentially due to a lower stability of the mAnxA2 surface. It should be noted that the far-UV CD spectrum of mAnxA2, when compared to wt AnxA2, indicated that the overall helical secondary structure was conserved, while the thermal denaturation profiles indicated small structural perturbations in mAnxA2 due to the mutation of six exposed amino acids that could explain the differences in interaction with NS5B [25]. The lower amount (1041 RU) of mAnxA2 immobilized to the chip compared to AnxA2 (3595 RU) was attributed to the immobilization procedure and the lower content of lysine residues in mAnxA2, having 4 out of 32 lysine residues replaced by serine (i.e. K308S, K309S and K310S, K313S). However, the lower surface density and stability have no practical consequence for the interpretation of the data.

Non-linear regression analysis of the sensorgrams for the interaction between NS5B and AnxA2 showed that it could not be well described by any simple mechanistic model, preventing the determination of kinetic parameters. Instead, an approximate steady state analysis of the SPR sensorgrams was performed in order to quantify the affinity for the interaction (Fig. 1e-h). The apparent dissociation constants (K_D_^app^) (Table 1) are thus approximations of the affinity.

**Table 1:** Steady state data for interaction between NS5B and immobilized AnxA2

Surface	*K*_D_^app^ (nM)	R_max_ (RU)
AnxA2	30	6200
mAnxA2	240	3260

Mean values from two experiments. Data from Fig. 1a and d

The mean K_D_^app^ for the interaction between AnxA2 and NS5B, based on two independent multicycle experiment where AnxA2 was immobilized and NS5B was run as analyte, was 30 nM. In the reverse experiment, the concentration of AnxA2 was too low relative to the K_D_^app^ for the affinity to be estimated.

### AnxA2 does not interact directly with NS3 or NS3/4A

To investigate if AnxA2 also interacts directly with the HCV NS3 protease, NS3 or the NS3/NS4A complex were run as analytes over sensor surfaces with immobilized AnxA2. However, no interactions were detected with either the NS3 protein at concentrations up to 270 nM or with the NS3/NS4A complex up to 500 nM (Additional file 1: Figure S1).

As a control, the effect of AnxA2 on the activity of NS3 protease was tested using a FRET-based enzyme activity assay, although no direct interaction was detected between NS3 and AnxA2 using the SPR assay. No effect on the enzymatic activity was detected when AnxA2 was present in a 5 to 1 M ratio to NS3 (Additional file 1: Figure S2, Table S1). The two different experimental approaches both indicate that AnxA2 does not interact directly with the NS3 protein of HCV.

### Influence of allosteric NS5B inhibitors on the AnxA2-NS5B interaction

To obtain an understanding of the functional implications of the interaction between AnxA2 and NS5B, we investigated the effect of allosteric NS5B inhibitors on the interaction.

When injecting AnxA2 over an NS5B surface, alone or in the presence of 500 nM filibuvir it was evident that filibuvir had little, if any, effect on the interaction (Fig. 2c). However, when filibuvir was injected over an NS5B-AnxA2 surface, it resulted in a significantly lower binding response (Fig. 2b), compared to when injected over an NS5B surface (Fig. 2a). When injecting NS5B concomitantly with filibuvir over immobilized AnxA2, a significantly reduced binding of NS5B to AnxA2 was also observed (Fig. 2d). This was not seen when performing the corresponding experiment with the polymerase inhibitor dasabuvir (Fig. 2d). These results show that the binding of filibuvir to NS5B impairs the interaction between NS5B and AnxA2. Reciprocally, when NS5B has formed a complex with AnxA2, its ability to interact with filibuvir is impaired (Table 2). However, the binding of AnxA2 to NS5B is not affected when NS5B is subjected to AnxA2 and filibuvir simultaneously, possibly attributable to a kinetic effect.

**Table 2:** Steady state data for filibuvir interacting with immobilized NS5B, and effect of AnxA2 on the interaction

Surface	*K*_D_^app^ ± SE (nM)		R_max_ ± SE (RU)	
NS5B^a^	160	±20	39	± 1.0
NS5B^b^	250	± 30	33	± 0.7
NS5B pre-injected with AnxA2	390	± 54	16	± 0.6

^a^Activated and inactivated blank reference surface

^b^Inactivated NS5B reference surface. SE = standard error. Data from Fig. 2a and b

### Influence of AnxA2 on the polymerase activity of NS5B

**Fig. 3:**
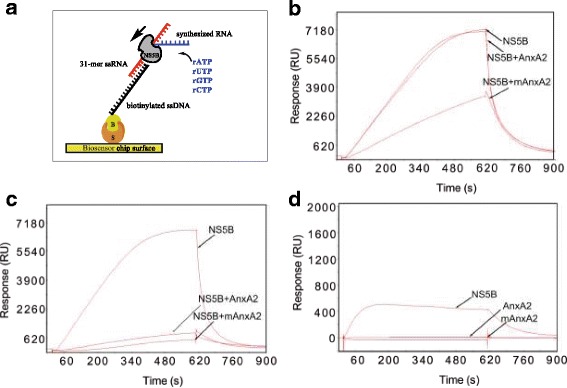
Effect of AnxA2 and mAnxA2 on the polymerase activity of NS5B, measured as the de novo synthesis of RNA using RNA-DNA hybrid surfaces and rNTPs. **a** Illustration of the biosensor-based HCV polymerase assay monitoring the de novo synthesis of RNA using RNA-DNA hybrid surfaces and rNTPs. A biotinylated (**b**) ssDNA oligo is captured on a biosensor chip via streptavidin (S) immobilized by amine coupling. The 31-mer RNA is subsequently hybridized to the ssDNA. 100 nM NS5B (**b** and **c**) and nucleotides are injected over the surface and the polymerase activity monitored in real-time in the presence of 100 nM (**b**) or 200 nM (**c**) AnxA2 or mAnxA2 (**d**) Binding of 100 nM NS5B, 1000 nM AnxA2 and 200 nM mAnxA2 to the sensor surfaces in the absence of rNTPs (negative control experiment)

The potential effect of AnxA2 on the polymerase activity of NS5B was investigated using an SPR-de novo polymerase assay, in which a 31-mer RNA, designed to be used for de novo polymerase assay [22] was captured on a partly complementary ssDNA immobilized on a sensor chip, and NS5B and rNTPs were injected as analytes in the absence or presence of AnxA2 (Fig. 3a).

The assay monitors the formation of the complementary RNA chain or nucleotides incorporation in real time. Reference experiments included the control experiment in which NS5B was injected alone, i.e. without rNTPs or AnxA2, or in which AnxA2 and mAnxA2 were injected without NS5B.

NS5B interacted with the 31-mer RNA (Fig. 3b-d). In contrast, neither AnxA2 nor mAnxA2 were observed to interact with the 31-mer RNA at concentrations up 1000 nM or 200 nM, respectively (Fig. 3d). The activity of NS5B was not affected by the presence of AnxA2 at equimolar amounts (Fig. 3b). However, when AnxA2 was added at a 2:1 M ratio to NS5B, the nucleotide incorporation rate was significantly reduced (Fig. 3c). mAnxA2 reduced the polymerase activity of NS5B more efficiently than AnxA2 (Fig. 3b, c).

### AnxA2 interactions with RNA

**Fig. 4:**
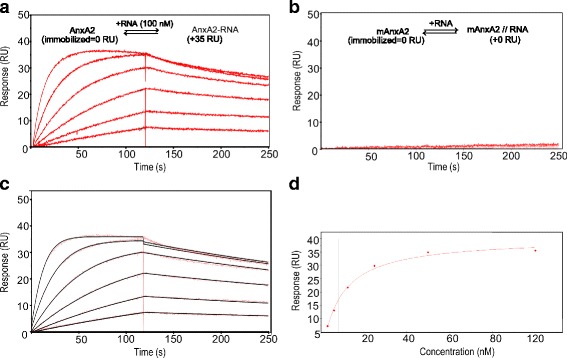
Interaction between the 8-mer RNA and AnxA2. The 8-mer RNA was injected at 3 to 100 nM over **a** immobilized AnxA2 (3595 RU) and **b** immobilized mAnxA2 (1041 RU). All sensorgrams were corrected for unspecific binding by blank and reference surface subtraction. An activated and inactivated surface was used as a reference. **c**) Non-linear regression analysis of the SPR sensorgram in (**a**) using a reversible 1-step 1:1 model. **d** Steady state analysis of report points taken at the end of the injections in (**a**). Vertical lines on steady-state analysis plots indicate K_D_ values

It was unexpected that AnxA2 did not interact with the 31-mer RNA captured by ssDNA (Fig. 3d). To confirm that the AnxA2 used in our experiments was functional and could bind RNA, a control experiment was performed. As it has been shown that AnxA2 has an intrinsic poly(G)-binding activity [25], an 8-mer RNA (5’GGG GAU UG-3′) containing a poly(G) sequence was injected as analyte over immobilized AnxA2 (Fig. 4a). It was confirmed that AnxA2 interacted with this RNA (Fig. 4a), although it was not able to bind to the immobilized 31-mer RNA (Fig. 3d). The K_D_ was found to be in the low nanomolar range, as determined by steady state affinity analysis and by global kinetic fit using a 1:1 model (Fig. 4c-d, Table 3). As a control, it was confirmed that the 8-mer RNA did not interact with the mAnxA2 surface (Fig. 4b).

**Table 3:** Steady state and kinetic data for the interaction between AnxA2 and the 8-mer RNA

Data analysis	*k*_a_ × 10^6^ (1/Ms)	*k*_d_ (1/s)	*K*_D_ (nM)	R_max_ (RU)
Steady state analysis	–	–	8.3	46
Kinetic analysis	0.99	0.002	2.0	35

Data from Fig. 4

### The interplay between AnxA2, NS5B and RNA

**Fig. 5:**
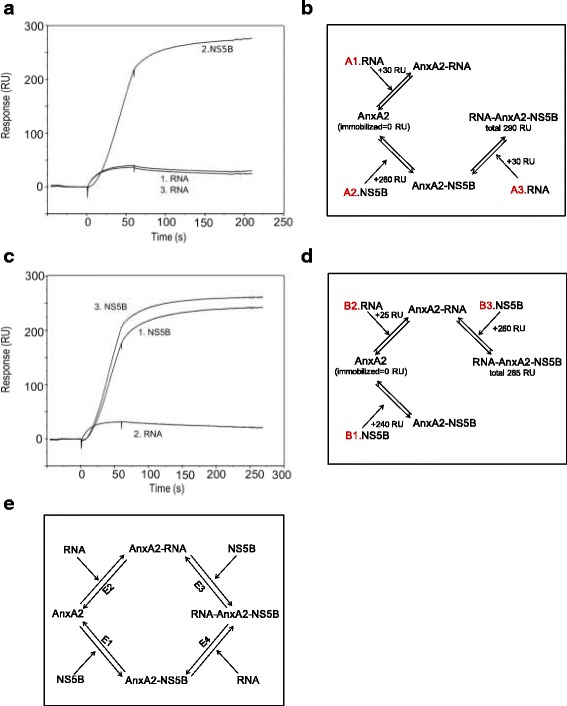
Analysis of the interplay between NS5B, AnxA2 and RNA determined by analysis of ternary complex formation using NS5B and the 8-mer RNA as analytes, and AnxA2 surfaces (3595 RU). The sensorgrams are aligned with respect to the baseline signal at the time for injection. **a** 1. Injection of 50 nM 8-mer RNA over an AnxA2 surface, providing a reference value for the RNA binding capacity of immobilized AnxA2. 2. 50 nM NS5B injected over the regenerated AnxA2 surface, resulting in a stable AnxA2-NS5B complex (as indicated by a stable new baseline after injection). 3. Injection of 50 nM 8-mer RNA over the AnxA2-NS5B surface. **c** 1. Injection of 50 nM NS5B over an AnxA2 surface, providing a reference value for the NS5B binding capacity of immobilized AnxA2. 2. 100 nM 8-mer RNA injected over the regenerated AnxA2 surface, creating a stable AnxA2-RNA complex. 3. 50 nM NS5B injected over the AnxA2-RNA complex surface.  **b** and **d** Schematic illustration of the experiments in  **a** and  **c**, respectively. **e** Schematic model of the interplay between NS5B and AnxA2 based on the presented data demonstrating formation of binary and ternary complexes, and where the 8-mer RNA interacts with AnxA2. Equilibrium 1 (E1) illustrates that AnxA2 can bind NS5B with strong affinity to form a stable binary complex. It has reduced NS5B polymerase activity and ability to interact with the allosteric inhibitor filibuvir. (E2) illustrates the interaction between AnxA2 and the 8-mer RNA, forming a binary complex that can interact with NS5B (E3), forming a ternary complex. This ternary complex can also be formed by the binding of the 8-mer RNA by the binary AnxA2-NS5B complex (E4). NS5B does not bind either the 8-mer RNA or the 31-mer RNA while in complex with AnxA2

To visualize the interplay between AnxA2, the 8-mer RNA and NS5B, the formation of a ternary complex between the three entities was explored using surfaces with immobilized AnxA2 and then injecting either 8-mer RNA or NS5B to form a stable binary complex, followed by injection of the third binding partner, thus potentially forming a ternary complex. Eight-mer RNA (50 nM) was first injected over AnxA2, providing a reference point for RNA-binding to AnxA2 (Fig. 5a). After regeneration of the surface, NS5B (50 nM) was injected over AnxA2, resulting in a stable AnxA2-NS5B complex, as indicated by a stable new baseline after injection (Fig. 5a). The injection of the 8-mer RNA over the AnxA2-NS5B surface resulted in the same signal as when 8-mer RNA was injected over AnxA2 alone (Fig. 5a). This suggests that 8-mer RNA binds to the surface via AnxA2, not NS5B (Fig. 5b).

To confirm this interpretation, the opposite experiment was performed, i.e. NS5B (50 nM) was injected over AnxA2 alone to get a reference value. Eight-mer RNA (100 nM) was then injected over the regenerated AnxA2 surface, creating a stable AnxA2-RNA complex (Fig. 5c). NS5B was subsequently injected, resulting in NS5B binding to the AnxA2-RNA complex. This injection of NS5B over the AnxA2-RNA complex only resulted in a slightly higher signal than for NS5B binding to AnxA2 alone (Fig. 5c). This confirms the formation of an RNA-AnxA2-NS5B complex (Fig. 5d).

Finally, injection of the 8-mer RNA and NS5B concomitantly (in ratio 1:1 or 2:1) over AnxA2 was compared to the response observed when injecting NS5B and the 8-mer RNA separately (data not shown). The response seen for the NS5B-RNA complex was identical to that resulting from the injection of NS5B alone, suggesting that the binding of the NS5B complex to the 8-mer RNA is disrupted upon NS5B binding to AnxA2.

## Discussion

In the present study we used a sensitive, time-resolved, biophysical real-time method to investigate the proposed interactions between AnxA2 and HCV polymerase NS5B and serine protease NS3 in more detail. We could not confirm any direct interactions between AnxA2 and NS3 or the NS3/NS4A complex, or an influence of the presence of AnxA2 upon NS3 protease activity (data not shown). This may indicate that the previously observed link between AnxA2 and NS3/NS4A [3, 4] is indirect, and a result of co-localization in the replication complex rather than of a direct and stable interaction between the two proteins. However, the previously described co-precipitation experiments [3] with AnxA2 and NS5B, were confirmed. We further characterized the kinetics and effects of allosteric inhibitors and specific RNA on the interaction. Intriguingly, our overall data revealed interplay between AnxA2, NS5B and specific RNA, which were found to create a stable ternary complex (illustrated in Fig. 5e).

Specifically, we demonstrated that AnxA2 and NS5B interact directly with strong affinity (Fig. 5e, E1), having a K_D_ value in the nanomolar range, and with very slow dissociation kinetics, possibly indicating conformational changes. A practical consequence of the high stability of the complexes was that the sensor surfaces had to be regenerated using a relatively harsh procedure. Thus, visual inspection of the sensorgrams for the interaction between AnxA2 and NS5B, and data showing the effect of allosteric inhibitors on the interactions, suggest that it involves a conformational change in one or both of the two proteins, induced upon binding. Additional complexities prevented the identification of a suitable mechanistic model for global regression analysis of the sensorgrams, precluding an in depth mechanistic and quantitative kinetic analysis. Notably, the interaction between AnxA2 and NS5B interfered with the ability of NS5B to interact with the allosteric inhibitor filibuvir significantly. It also impaired the polymerase activity of NS5B seen by a significantly drop of the nucleotide incorporation rate.

In the literature, AnxA2 is described as an RNA binding protein [19, 26], but the previously published binding to RNA, as measured by SPR, was only seen for concentrations higher than 500 nM [19]. By SPR, a strong binding of immobilized AnxA2 to the 8-mer RNA was observed, of low nanomolar affinity (Fig. 5d, E2). However, no binding of AnxA2 to the 31-mer RNA captured by DNA immobilized on the SPR biosensor chip was observed. The RNA sequence used was originally constructed for a fluorescent based de novo polymerase activity assay [22]. To optimize fluorescence detection, this RNA construct was designed to minimize secondary structure formation. The fact that the 31-mer RNA does not bind AnxA2, suggests that the interaction between AnxA2 and RNA is dependent on a specific RNA sequence in combination with a higher order structure. This is in line with previous experiments, which showed that binding of AnxA2 to mRNA is dependent on a specific secondary RNA structure [27]. On the other hand, the 8-mer RNA, which similarly to the 31-mer RNA does not contain any higher order structure, binds strongly to AnxA2. However, it has been shown that AnxA2 has an intrinsic poly(G)-binding activity [25]. Since the 8-mer RNA contains a poly(G) sequence, this is a likely explanation for its binding to AnxA2. The 31-mer RNA does not contain a poly(G)-binding site nor a AA(C/G)(A/U)G consensus sequence also reported important for RNA binding to AnxA2 [28]. As expected, the engineered mAnxA2 (see Materials & Methods) variant did not bind to either RNAs [19].

The presented analysis of the interactions and interplay between AnxA2, NS5B and RNA should bear in mind that the experiments were performed in vitro using a biosensor and isolated proteins in a biological buffer system. Although we have attempted to simulate physiological conditions, it is clearly a simpler system than the corresponding system in vivo**.** However, the advantage of the current approach is that we can isolate certain interactions and study them in detail, without confounding factors. It is possible that the interactions and the characteristics we observe in vitro are modulated in an in vivo system. For example, viral replication may require that AnxA2, or the complex, also interacts with specific lipids to ensure that this process occurs at the correct site. To obtain a better understanding of the biological relevance and specificity of RNA binding to NS5B and AnxA2 in vivo, it would be of interest to base future studies on designed RNA constructs derived from the HCV RNA genome. Furthermore, human AnxA2 should ideally be used instead of bovine AnxA2, although the protein is highly conserved and the mammalian species only differ by a few amino acids [6].

The studies investigating the interplay between AnxA2, NS5B and RNA showed that binding of AnxA2 to NS5B did not prevent AnxA2 from binding to the 8-mer RNA simultaneously (Fig. 5e, E3-E4). This indicates that the AnxA2 RNA binding site is localized at a different site than the NS5B binding site. This was also supported by the ability of the non-RNA binding mutant mAnxA2 to interact with NS5B. The interaction between mAnxA2 and NS5B was weaker and the complex dissociated more rapidly than for the *wt* AnxA2. However, the non-ideal shape of the sensorgrams indicated that the interaction was not stable, potentially due to a low stability of the mAnxA2 surface, which can be explained by structural perturbations introduced by mutating AnxA2, indicated by a lower apparent transition temperature for mAnxA2 (~48 °C) than the native form (~55 °C) [19]. However, previous biophysical studies have revealed the preservation of the overall α-helical structural integrity of the mAnxA2 [19]. Due to the apparent unstable interaction between the non-RNA binding mAnxA2 and NS5B, it is not possible to infer whether the mAnxA2 binds with a lower affinity to NS5B than *wt* AnxA2.

NS5B, like AnxA2, interacts with RNA [21]. However, in contrast to AnxA2 in complex with NS5B, the interplay studies showed that NS5B could not bind the 8-mer RNA when in complex with AnxA2 (Fig. 5e, E4). Furthermore, the lack of interaction with RNA when in complex with AnxA2 is likely explaining the observed reduced polymerase activity of NS5B, resulting in decreased rNTP incorporation rate.

Previous studies of the role(s) of AnxA2 in the life cycle of HCV have suggested that AnxA2 may play an important role in several processes, ranging from replication complex formation to virus particle assembly [3, 5]. It was previously demonstrated that silencing of the expression of AnxA2 has no effect on HCV viral RNA replication but resulted in a significant reduction of virus titers [5, 29]. Based on this, they suggested that AnxA2 plays a role in HCV assembly rather than in genome replication or virion release. Another study proposed that AnxA2 recruits HCV NS proteins and causes their enrichment on lipid rafts to form the HCV replication complex, since AnxA2 is known to induce the formation of the lipid raft microdomains [3]. Our data show that the NS5B polymerase activity is reduced when NS5B interacts with AnxA2 and that NS5B is not able to bind RNA when in complex with AnxA2, implying that the functional role of this interaction is not related to events in the viral life cycle requiring an active NS5B polymerase. This supports the hypothesis that AnxA2 plays a role in HCV replication complex assembly rather than in genome replication. Interestingly, also another annexin, AnxA3, was more recently found required for efficient HCV particle production, thus suggesting a more general role for the Annexins in the HCV viral life cycle [30]. Another possibility is that NS5B uses AnxA2 to transport viral RNA together with host mRNA as it has been shown that AnxA2 is involved in the transport of c-*myc* mRNA to the perinuclear region [31]. However, it is also possible that AnxA2 binds to HCV RNA in vivo, as has been shown for an RNA of the infectious bronchitis virus. In the latter case, it binds to a pseudoknot structure and reduces the −1 ribosomal frameshifting efficiency important for viral replication [32]. In this manner, AnxA2 may have a function in the host defense against certain viruses.

Taken together, it appears that the binding of AnxA2 to NS5B reduces the inherent and important structural flexibility of NS5B and locks the protein in a conformation, with impaired ability to interact with both substrates and inhibitors. This is interesting from a HCV drug discovery perspective as novel drugs may be designed with a similar mode of action, potentially targeting the AnxA2-NS5B interaction interface and thus preventing NS5B polymerase activity. To be able to understand how to potentially modulate, stabilize or mimic the interaction between AnxA2 and NS5B to inhibit NS5B polymerase activity by a small molecular drug, it is of relevance to further elucidate the structural details of this interaction both with respect to structural conformation of the proteins involved and information of the exact protein regions important for binding.

The stable complex formation between AnxA2 and HCV NS5B, in this study shown to influence the catalytic activity of NS5B and its sensitivity to allosteric inhibitors, may indeed also disturb normal cellular functions of AnxA2, for example its role in mRNA transport and translation in the host cell [28]. Interactions of viral proteins with host proteins that have key regulatory functions in normal cell growth were previously found to set the stage for carcinogenesis by interfering with cellular proliferation or cell cycle checkpoints functioning to maintain genomic integrity (reviewed in [33]). Furthermore, the AnxA2-NS5B interaction may also be involved in the HCV-associated pro-inflammatory milieu connected to HCC development [29, 34–36]. The direct interaction between HCV NS5B and the host factor AnxA2 shown here may thus be an important link between HCC development and the HCV viral life cycle.

## Conclusions

The biophysical approach taken in this study was suitable for investigating direct interactions between two non-structural proteins of HCV (NS5B and NS3), AnxA2, RNA and filibuvir. It was possible to elucidate the complex interplay between these molecular species and confirmed interactions between AnxA2 and NS5B, but not with NS3. The data suggest that AnxA2 may have an important role in HCV infections and the development of HCC. This is relevant also for design of new allosteric inhibitors targeting NS5B.

## Additional file

Additional file 1:
**Figure S1.** No Interactions between AnxA2 and NS3 or NS3/4A were observed by SPR. **Figure S2.** No Influence of AnxA2 upon enzyme activity of NS3. **Table S1.** Michaelis constant (KM) and maximal velocity (Vmax) for NS3 enzyme activity with and without the presence of AnxA2, derived from a FRET enzyme activity assay (Figure S2). (DOC 237 kb)

## Abbreviations

AnxA2: Annexin A2
DAAs: Direct acting antivirals
DTT: Dithiothreitol
EDTA: Ethylene diamine tetra acetic acid
ER: Endoplasmic reticulum
HCC: Hepatocellular carcinoma
HCV: Hepatitis C virus
HEPES: 4-(2-Hydroxyethyl)-1-piperazineethanesulfonic acid
*k*_a_: Association rates
K_D_: Dissociation constants
*k*_d_: Dissociation rates
mRNP: Messenger ribonucleoprotein
NS: Non-structural
NS5BΔ21: Ectodomain of NS5B
R_max_: Maximum binding responses
rNTPs: Ribo nucleotide triphosphates
RU: Refractive units
SCK: Single cycle kinetic
SPR: Surface plasmon resonance
wt: Wild type
