# Supplementary material for: Characterization of interactions between hepatitis C virus NS5B polymerase, annexin A2 and RNA – effects on NS5B catalysis and allosteric inhibition
Source: Virol J. 2017 Dec 11;14:236. doi: 10.1186/s12985-017-0904-4 (PMC5725786; doi:10.1186/s12985-017-0904-4)
Supplement: Additional file 1: — Figure S1. No Interactions between AnxA2 and NS3 or NS3/4A were observed by SPR. Figure S2. No Influence of AnxA2 upon enzyme activity of NS3. Table S1. Michaelis constant (KM) and maximal velocity (Vmax) for NS3 enzyme activity with and without the presence of AnxA2, derived from a FRET enzyme activity assay (Figure S2). (DOC 237 kb) [file 12985_2017_904_MOESM1_ESM.doc]

# Supplementary data

# Characterization of interactions between hepatitis C virus NS5B polymerase, annexin A2 and RNA – Effects on NS5B catalysis and allosteric inhibition

**Sara M. Ø. Solbak^1^, Eldar Abdurakhmanov^1^, Anni Vedeler^2^ and U. Helena Danielson^1, 3*^**

^1^Department of Chemistry – BMC, Uppsala University, Uppsala, Sweden

^2^Department of Biomedicine, University of Bergen, Bergen, Norway.

^3^Science for Life Laboratory, Uppsala University, Uppsala, Sweden

*****Correspondence: [helena.danielson@kemi.uu.se](mailto:helena.danielson@kemi.uu.se); Tel.: +46-18-4714545

Sara.Solbak@sund.ku.dk; eldar.abdurakhmanov@kemi.uu.se; Anni.Vedeler@uib.no; [helena.danielson@kemi.uu.se](mailto:helena.danielson@kemi.uu.se)

**Figure S1.** No evidence for interactions between AnxA2 and NS3 or NS3/4A was found by SPR biosensor analysis. Sensorgrams for injection of 34 to 268 nM NS3 over immobilized 769 RU AnxA2 (A) or 31 to 500 nM NS3/NS4A over immobilized 5154 RU AnxA2 (B).

**Figure S2.** AnxA2 does not affect HCV NS3 protease activity. Plots of initial rate versus substrate concentration for the hydrolysis of a FRET peptide by HCV NS3 protease in the absence and presence of different ratios and variants of AnxA2: A) NS3 alone. B) NS3 in the presence of AnxA2 (1:1). C) NS3 in the presence of AnxA2 (1:5). D) NS3 in the presence of mAnxA2 (1:1).

**Table S1.** Michaelis-Menten constants (K_M_) and maximal velocities (V_max_) for NS3 protease catalysis in the absence and presence of AnxA2, derived from a FRET-based enzyme activity assay (data from Figure S2).

| Sample | V_max_ | K_m_ | |
| --- | --- | --- | --- |
| NS3 | 0.0022* |  | 1.93* |
| NS3:ANXA2 (1:1) | 0.0025 |  | 2.46 |
| NS3:ANXA2 (1:5) | 0.0028 |  | 1.56 |
| NS3: mANXA2 (1:1) | 0.0029 |  | 1.54 |

*Mean of three individual measurements
